# Body Weight Cycling with Identical Diet Composition Does Not Affect Energy Balance and Has No Adverse Effect on Metabolic Health Parameters

**DOI:** 10.3390/nu9101149

**Published:** 2017-10-20

**Authors:** Inge F. Palm, Rianne G. A. E. Schram, Hans J. M. Swarts, Evert M. van Schothorst, Jaap Keijer

**Affiliations:** Human and Animal Physiology, Wageningen University, P.O. Box 338, 6700 AH Wageningen, The Netherlands; inge.palm@wur.nl (I.F.P.); rianne.schram@wur.nl (R.G.A.E.S.); hans.swarts@wur.nl (H.J.M.S.); evert.vanschothorst@wur.nl (E.M.v.S.)

**Keywords:** body weight cycling, nutritional physiology, basal metabolism, C57BL6, serum marker, adipose tissue, yo-yo

## Abstract

Background: Body weight (BW) cycling, the yo-yo effect, is generally thought to have adverse effects on human metabolic health. However, human and animal experiments are limited in number and do not provide clear answers, partly due to large variations in experimental design, parameters measured, and definitions of BW cycling. Here, we examined the effect of repetitive BW cycling versus single- and non-cycling control groups, without alterations in diet composition, on steady state BW and metabolic parameters. Methods: We induced well-defined BW cycles on a semi-purified high fat diet in C57BL/6J mice, a well-described animal model for diet-induced obesity, and measured energy expenditure and relevant metabolic parameters. Results: Our setup indeed resulted in the intended BW changes and always reached a stage of energy balance. A history of weight cycling did not result in increased BW or fat mass compared with the control group, nor in deteriorated serum concentrations of glucose, adipokines and serum triglyceride and free fatty acid (FFA) concentrations. If anything, BW tended to be reduced, presumably because of a reduced overall energy intake in BW cycling animals. Conclusion: Repeated cycling in BW without changes in diet composition does not lead to impaired metabolic health nor increased BW (gain).

## 1. Introduction

The prevalence of obesity continues to rise worldwide. Obesity is the result of a long-term positive energy balance, where energy intake exceeds energy expenditure (EE). It is generally not very hard to turn this balance into a negative one to lose body weight (BW). However, maintaining a lower BW appears to be very difficult [1,2], and lost BW is often regained. Individuals attempting weight loss often show repeated cycles of intentional BW loss and unwanted BW regain. This BW cycling is also known as the yo-yo effect.

Popular wisdom says that BW cycling in humans negatively affects (metabolic) health. Abundant warnings can be found on the internet. These warnings concern adverse effects on BW and on health. It is even suggested that it is better to remain obese than to BW cycle [3]. Despite an abundance of statements warning against the negative effects of weight cycling, little substantiating scientific proof exists.

A search in Pubmed for “weight cycling” in the title/abstract yields 324 hits of which 311 are in English and published between 1981 and 2017. This is 0.2% of the number of publications on “obesity” (193,371 hits of which 175,214 are in English). Combining the two search terms yields 118 hits in English (on 4 August 2017). These numbers indicate that the scientific focus within the field of obesity research is not on the mechanisms and consequences of BW cycling, despite strong public opinions about this phenomenon.

In 1994, the National Task Force on the prevention and treatment of obesity of the National Institutes of Health (USA) published a review on possible adverse effects of BW cycling [3]. They concluded that there is little support for adverse effects of BW cycling on metabolism, but that firm conclusions on the (positive or negative) health consequences of BW cycling were not possible due to diverse and frequently unclear definitions and assessment methods resulting in a large variation in the results.

Around the same time, in 1993, a review of animal literature on BW cycling was published [4]. Evaluating 24 animal studies performed between 1973 and 1993, the conclusion was that very few data actually support adverse effects of BW cycling on BW and adiposity, EE, lipid metabolism, or insulin sensitivity. Approaches to induce BW cycles were diverse, including cycles ranging from 2 days to 7 weeks, cycles induced by fasting and re-feeding, by changing from a high fat to a low fat diet, by a calorie restriction on the same diet, or by a combination of both calorie restriction and diet change [5]. In addition, proper age-matched controls were often lacking [4]. After this review, only a limited number of animal studies on BW cycling were published (40 with ‘Weight cycling’ in title/abstract, Pubmed), again with little consistency in the animal models used (species, knockouts, strains, sex), the methods to induce BW cycles, and the parameters that have been measured. Again, BW cycles did not affect various metabolic parameters [6], or increased BW [7,8], or decreased BW and fat mass [9]. In other words, very inconsistent and conflicting data also exist in more recent literature, due to the variety of approaches that are used to tackle this issue.

In summary, negative effects of BW cycling are firmly embedded in public opinion, but the existing published data on humans and animals do not allow for firm conclusions about the (metabolic) health consequences of BW cycling. This is at least partly due to the large variety in experimental approaches; e.g., BW cycles are often induced by a change between diets [10,11,12,13,14], and by the use of non-purified diets (all studies, except [13,14]), in aging animals [12], or in animals of unknown age [11], or do not include multiple cycles [13,14]. In addition, weight cycles are used that are too short to mimic human BW cycling [15,16]. Finally, a wide variety of animal models is used, and not all studies are aimed at metabolic assessment [17].

Thus, a well-controlled intervention study that selectively investigates the effect of repetitive BW cycling on BW changes irrespective of dietary habits, and monitors in vivo metabolic status, is clearly needed. Therefore, we designed a straightforward animal experiment with a well-defined semi-purified diet and clearly defined BW cycles, to answer the question: Does a history of BW cycling affect metabolic health? We used an animal model widely used for diet-induced obesity, the C57BL6/J strain of mice. To improve the present study compared with previous animal studies, we assessed whole-body energy metabolism using indirect calorimetry, and combined the data with changes in BW and food intake throughout the different phases of the study. In addition, we measured a number of relevant metabolic parameters (adiposity; adipocyte size; and serum glucose, insulin, leptin, adiponectin, and free fatty acids (FFA)) in fasted animals at the end of the study.

## 2. Materials and Methods

### 2.1. Animals and Housing

Nine-week-old male C57BL\6JOlaHsd mice were obtained from Harlan (Horst, The Netherlands). Upon arrival, mice were housed individually in clear macrolon cages (type II) in a temperature- and humidity-controlled room (22 °C ± 2 °C and 50 ± 10%, respectively). They were provided with *ad libitum* food and water. The food was a semi-synthetic moderate-fat diet (MFD, 30 en % fat, Research Diet Services, Wijk bij Duurstede, The Netherlands), mimicking human fat intake, as used previously [18]. Lights were on from 6 to 18 h at a maximal intensity of 60 lux during the light period. After an ACCLIMATIZATION period of 2.5–3 weeks the experiments started when the animals were 12 weeks of age. The animal experiment was according to Dutch law and was approved by the animal ethical committee of the Wageningen University (DEC 2009076c).

### 2.2. Induction of Body Weight (BW) Cycling

**Figure 1:**
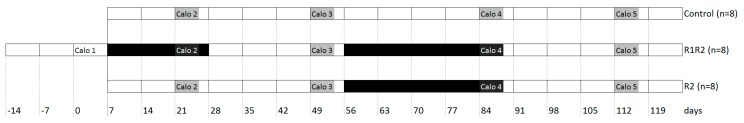
Study design. All mice were fed a semi-purified medium-fat diet (MFD) throughout the whole study. After 3 weeks of acclimatization, the first weight cycle was started at *t* = day 7 by 3 weeks of calorie restriction (black bars, until *t* = day 28), followed by 4 weeks of *ad libitum* food intake (white bars). The second weight cycle was started at *t* = day 56 with 5 weeks of calorie restriction (until *t* = day 91), followed by 5 weeks of *ad libitum* food consumption. Five indirect calorimetry measurements were done, starting at *t* = day 0, 21, 49, 84, and 112, and coded Calo1 to Calo5. Only the data of Calo5 are reported.

At *t* = day −5, animals were randomly divided into three groups of *n* = 8 each: a control group which received *ad libitum* food throughout the study (C), a group which underwent two BW cycles (R1R2), and a group that underwent only the second BW cycle (R2; Figure 1). BW cycles were induced by limiting the amount of food available to the animals to 80% of their individual *ad libitum* food intake in the previous two weeks without changing the diet, i.e., by calorie restriction (CR). The daily amount of food was weighed to within 0.1 g precisely for each individual animal, and provided daily at 16 h.

The first BW cycle (group R1R2) was started after three weeks of *ad libitum* intake of the MFD, at *t* = day 7. After three weeks on the restricted diet (*t* = day 28), animals were allowed to eat *ad libitum* for four weeks. During this BW cycle, group R2 received the MFD *ad libitum*. The second BW cycle was started after the 4 week *ad libitum* period of the R1R2 group (*t* = day 56). This time, both the R1R2 and the R2 groups received the 20% food restriction, in the same way as during the first BW cycle. This restriction was continued until the BW of the animals stabilized (i.e., did not change over 1 week, or changed in parallel with the control group). After 5 weeks on restriction (*t* = day 91), R2 and R1R2 animals were allowed to eat *ad libitum* to regain BW until BW stabilized (also for a total duration of 5 weeks).

### 2.3. Parameters of Interest

Throughout the study, the following parameters were repeatedly measured: BW, food intake (FI), and energy expenditure (EE). BW and FI were measured each Monday and Friday throughout the whole study. During indirect calorimetry measurements, BW and FI were also measured on Wednesdays. BW was measured using the same calibrated scale for all measurements. FI was calculated over 4 day (Monday to Friday) or 3 day (Friday to Monday) periods by weighing the amount of food supplied and the amount of food left in the home cage of the animals.

Throughout the study, in vivo EE was determined by indirect calorimetry (see below) in all mice: after the run-in period (i.e., one week prior to the first BW cycle), at the end of the first restriction period (*t* = day 21), at the end of the first *ad libitum* re-feeding period (*t* = day 49), at the end of the second restriction period (*t* = day 84), and at the end of the second *ad libitum* re-feeding period (*t* = day 112). These five measurements are coded Calo1 to Calo5 respectively (see Figure 1).

### 2.4. Indirect Calorimetry

Indirect calorimetry was performed using an open-circuit LabMaster Metabolic Research Platform (TSE Systems GmbH, Bad Homburg, Germany) with 12 cages. Oxygen consumption and CO_2_ production were measured continuously for 48 h. Only the last 24 h were used for analysis and calculations of EE and respiratory exchange ratio (RER), as described previously [13]. Per batch, four animals from each of the three groups were simultaneously measured.

### 2.5. End Point Parameters

To answer the research question: “Does a history of BW cycling affect metabolic health?” we compared the three experimental groups during the last *ad libitum* re-feeding period. For in vivo energy balance measurements, Calo5 data were used (*t* = day 112–116). For BW and FI, *t* = day 119 data were used, after Calo5 and prior to dissections. For tissue and serum analyses, data at dissection were used at *t* = day 126.

For dissection, all animals were fasted overnight (16 h), and subsequently anaesthetized with an isoflurane, oxygen, and nitrous oxide mixture. Blood was collected via orbital exsanguination into Mini collect serum tubes (Greiner Bio-one, Longwood, FL, USA), allowed to clot on ice for at least 1 h, and then centrifuged for 10 min at 3000× *g* at 4 °C. Serum was aspirated, and stored at −80 °C in aliquots of 50 µL maximum for later analysis.

Mice were killed by cervical dislocation and the body cavity was opened to harvest various tissues. White adipose tissue (WAT) was harvested from epididymal (eWAT), perirenal (periWAT), and mesenteric (mWAT) locations. All WAT depots were weighed except for mWAT. The eWAT and periWAT depots of the right side of the body were snap frozen in liquid nitrogen and stored at −80 °C. The depots of the other body side were fixed in 4% paraformaldehyde dissolved in 0.1 M PBS for 24 h at 4 °C, rinsed in 70% ethanol and directly embedded in paraffin for later histological analysis.

### 2.6. Serum Measurements

Serum concentrations were measured using commercial kits for free fatty acids (FFA, NEFA HR(2) kit, Wako Chemicals GmbH, Neuss, Germany) and triacylglycerides (TG, Triacylglycerides liquicolor kit, Human Diagnostics, Wiesbaden, Germany). All analyses were performed according to the manufacturers’ protocols, but the volumes were scaled down to allow for analysis with a plate reader (BioTek Synergy HT, Bad Friedrichshall, Germany).

Serum concentrations of insulin, leptin, and adiponectin were measured by Milliplex analysis (Millipore Corporation, Billerica, MA, USA). The sera were diluted 5 times (leptin, insulin) or 5000 times (adiponectin). The assays were conducted according to the manufacturer’s protocol and measured using the Bio-plex 200 system with Bio-plex manager software (Biorad Laboratories, Veenendaal, The Netherlands).

For glucose analysis, the sera were diluted 10 times in 0.3 M trichloroacetic acid (Merck, Darmstadt, Germany) and centrifuged for 5 min at 1750× *g*. The supernatant was mixed with glucose oxidase solution (1:5; GOD-PAP kit, Roche, Woerden, The Netherlands) in a 96-well plate and, after 30 min incubation at room temperature, the extinction at 490 nm was measured using a 96-well plate reader (BioTec Synergy HT, Bad Friedrichshall, Germany).

Serum samples were measured in duplicate and averaged, and concentrations were calculated using standard curves.

### 2.7. Histology of Epididymal Adipose Tissue

For the determination of adipocyte size, paraffin-embedded eWAT tissues were sectioned at 5 µm using a Reichert microtome (Reichert-Jung 2030, Cambridge Instruments, Heidelberg, Germany) and mounted on Superfrost plus slides (Menzel-Gläser, Menzel GmbH & Co. KG, Braunschweig, Germany). Sections were stained with PAS-Haematoxilin, resulting in blue-colored nuclei and purple cell membranes and cytoplasm. Tissues were examined under an Axioskop 2 microscope, and jpg images were acquired at 20× magnification using an Axiocam MR5 camera and AxioVision software v4.82 (Zeiss GmbH, Jena, Germany). Average adipocyte size was determined on digital images using AxioVision v4.82 (Zeiss GmbH, Jena, Germany), by drawing cell circumferences and calculating surface area in µm^2^ of 400 adipocytes per animal.

### 2.8. Data Analysis and Statistics

The in vivo metabolic state after BW cycling was assessed by calculating the energy balance (Energy Intake (EI) over EE) during the Calo5 measurements. Twenty-four-hour patterns and average values for VO_2_ (L/min), VCO_2_ (L/min), RER (VCO_2_/VO_2_), and EE (kcal/min) were analyzed. RER data were also analyzed using percentage relative cumulative frequency analysis (PRCF) [19]. This allows for systematic comparison of large datasets and is sensitive to detect small differences between groups.

Both absolute BW at *t* = day 119 and BW change from *t* = day 0 to *t* = day 119 were analyzed. FI was calculated as average FI (g/day), and was compared at *t* = day 119 and cumulative from *t* = day 0–119. Furthermore, we evaluated whether the second BW cycle induced comparable changes in the R1R2 and R2 groups, by comparing BW and FI changes during the restricted and *ad libitum* period. Food Efficiency (FE = ratio of BW gain over FI) was calculated for the different periods, and compared between the three groups.

All data are shown as mean ± standard error of the mean. Statistical analyses were performed using Graphpad Prism v5.04 (Graphpad Software Inc., San Diego, CA, USA). We did not observe differences between batches, so analyzed and present all data based on all animals. Data were checked for normality. Statistical analysis of all endpoint measurements was performed with a one-way ANOVA, followed by a Bonferroni post-hoc analysis when significant effects were detected by the ANOVA (*p* < 0.05).

## 3. Results

### 3.1. BW Cycling

**Figure 2:**
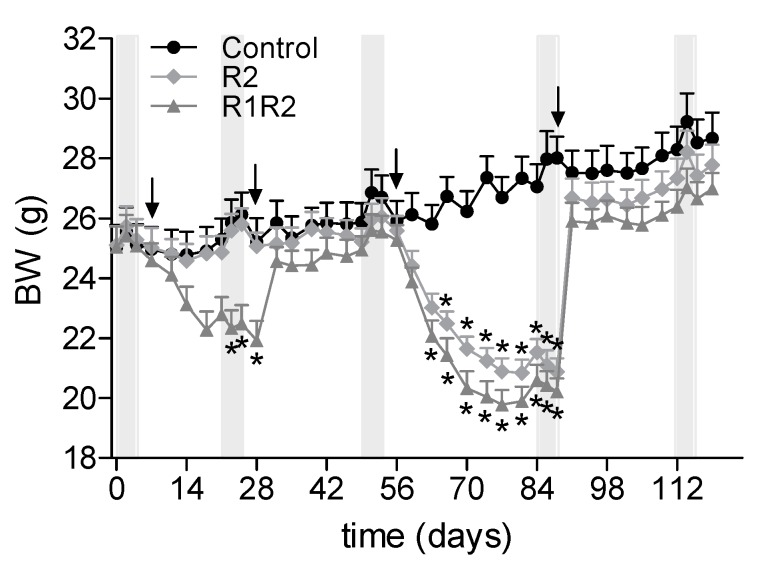
Body weight (BW) changes. BW of the three experimental groups are shown longitudinally. The grey bars indicate the periods of indirect calorimetry, and the arrows indicate the start of a calorie restriction or *ad libitum* refeeding phases. * indicate significant differences compared with the control group (*p* < 0.05).

The periods of calorie restriction (CR) and *ad libitum* re-feeding using identical diets resulted in clear BW cycles (Figure 2). The BWs at the start of the study were comparable between the groups. The calorie restriction resulted in immediate BW loss, which stabilized within the period of calorie restriction, during both the first and second BW cycle. BWs increased quickly after the start of the *ad libitum* re-feeding (Figure 2, 2nd and 4th arrows), and thereafter followed the *ad libitum* control group closely, but at a slightly lower mean BW in the two cycling groups. As intended by the design, the BW indeed stabilized during each restriction and re-feeding period, with stabilized being defined as not changing over 1 week, or changing parallel with the control group. As a result, all indirect calorimetry measurements were indeed performed during weight stable periods.

### 3.2. Effects of a History of BW Cycling

#### 3.2.1. Additional BW Cycle: R1R2 vs. R2

During the second BW cycle, BWs started to stabilize after two weeks of CR and remained stable for the following 3 weeks of CR in both cycling groups. The control group showed some growth during the same period. The amount of BW lost during the first 14 days of CR was significantly higher in the R1R2 group who received an earlier BW cycle, compared with the R2 group (−4.95 ± 0.32 g versus −3.94 ± 0.25 g, *p* < 0.0001). The BW recovery during ad lib re-feeding was comparable between the R2 and R1R2 groups: in the first three days BW increased rapidly to remain stable and parallel to the control group thereafter (Figure 2). Food intake during these first 3 days was significantly higher but comparable in both BW cycle groups (R1R2: 4.98 ± 0.20 g/day; R2: 4.94 ± 0.19 g/day), compared with the control group (2.65 ± 0.05 g/day, *p* < 0.0001). Food intake normalized to control concentrations thereafter and was comparable between all three groups. The two BW cycle groups gained the same amount of BW during the first 3 days of *ad libitum* re-feeding (R1R2: 5.7 ± 0.54 g; R2: 5.8 ± 0.42 g).

#### 3.2.2. BW, Food Intake, and Food Efficiency at the Endpoint

Total BW gain over the experimental timeframe differed significantly between groups; R1R2 animals gained the least BW, followed by R2 animals, whereas control animals gained the most BW (Table 1). Cumulative food intake over the whole experimental period (*t* = day 0–119) showed the same pattern: R1R2 animals ate the least, R2 animals were in between, and control animals ate the most. At the end of the study, i.e., *t* = day 116–119, the groups did not differ significantly from each other in absolute BW, nor in daily food intake, nor in food efficiency (Table 1).

#### 3.2.3. EE, RER, and Energy Balance at the Endpoint

In vivo measurements of oxygen consumption and carbon dioxide production were used to calculate RER and EE at different time points throughout the study. These parameters showed clear diurnal rhythms, with minimal concentrations during the light period, increasing concentrations towards the onset of the dark period, and maximal concentrations in the dark period. This pattern corresponded to the pattern in food intake (data not shown), with most food consumption occurring from just before the onset of the dark period into the first hours of the dark period.

**Figure 3:**
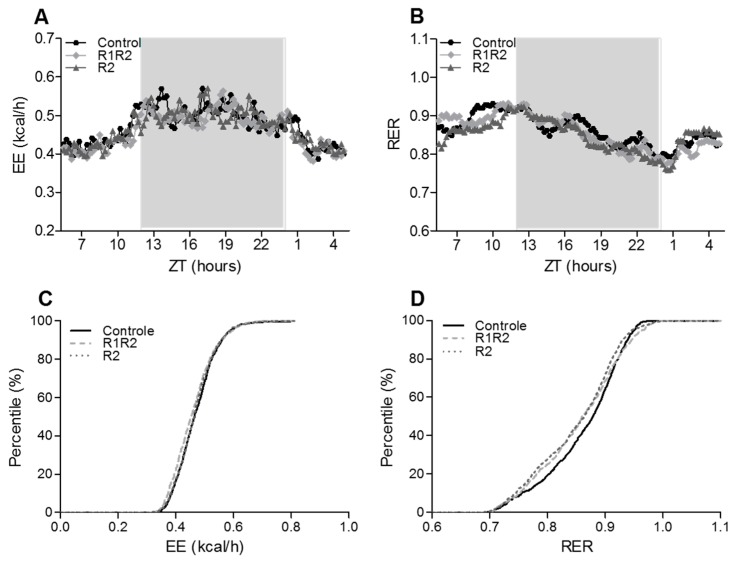
Energy metabolism. Panels (**A**,**B**): Diurnal patterns of energy expenditure (EE, panel (**A**)) and respiratory exchange ratio (RER, panel (**B**)) near the end of the study (*t* = day 112). Data are from the last 24 h of the 48 h measurement. Results are presented as means, without SEM for clarity of the figures. Time is represented as Zeitgeber Time (ZT), i.e., time relative to the light-dark schedule; ZT 0 = lights on, and ZT 12 = lights off. The grey area represents the dark period. Panels (**C**,**D**): Percentage relative cumulative frequency analysis (PRCF) curves for EE (panel (**C**)) and RER (panel (**D**)) during the last 24 h of the calorimetry measurement. No significant differences between any of the experimental groups were seen for any of the parameters.

Rhythmic patterns were comparable between experimental groups at the start of the study (Calo1, not shown), during the stable period after the first BW cycle (Calo3, not shown), and just before the end of the study after two BW cycles (Calo5, Figure 3A). PRCF analysis of the EE and RER data confirmed that the three experimental groups behaved in the same way; the EC50 value as well as the slope of the curves did not differ between experimental groups (Figure 3B). During the weight loss phase of each BW cycle (Calo2 and Calo4), rhythmic patterns looked different due to the fact that calorie-restricted animals were fed at a specific time point, and ate their meal immediately upon delivery at 2 h before the onset of the dark period. These results were therefore not further analyzed.

**Figure 4:**
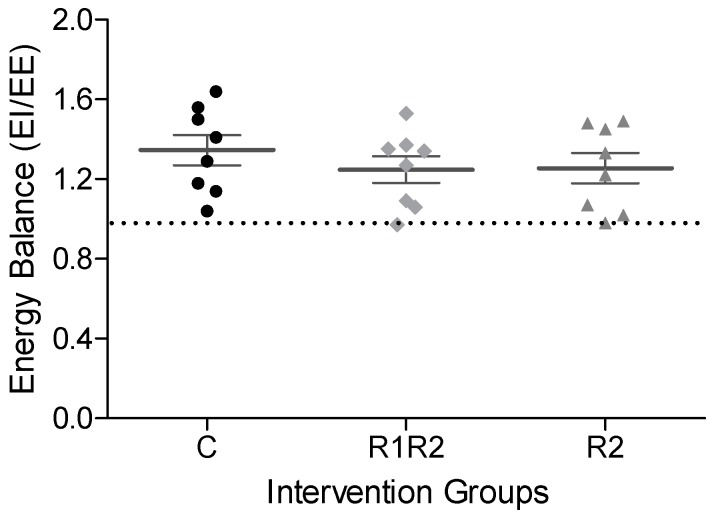
Energy Balance. Individual energy balance (energy intake (EI)/energy expenditure (EE)) during the last 24 h of the Calo5 measurement. The dotted line represents a ratio of 1, meaning that energy intake equals energy expenditure. For each group, individual data are shown in different symbols, and mean ± SEM are shown as horizontal lines with error bars. There were no statistical differences in energy balance between the groups.

Energy balance (EI/EE) values during the last 24 h of the Calo5 measurement were highly comparable between the three treatment groups (Figure 4). Calculating the energy balance over the total 48 h of the measurement allowed comparison with BW changes. As expected, a higher BW gain correlated with a higher energy balance with the same relation for the three experimental groups (data not shown).

#### 3.2.4. Adipose Tissue Weight and Histology

**Figure 5:**
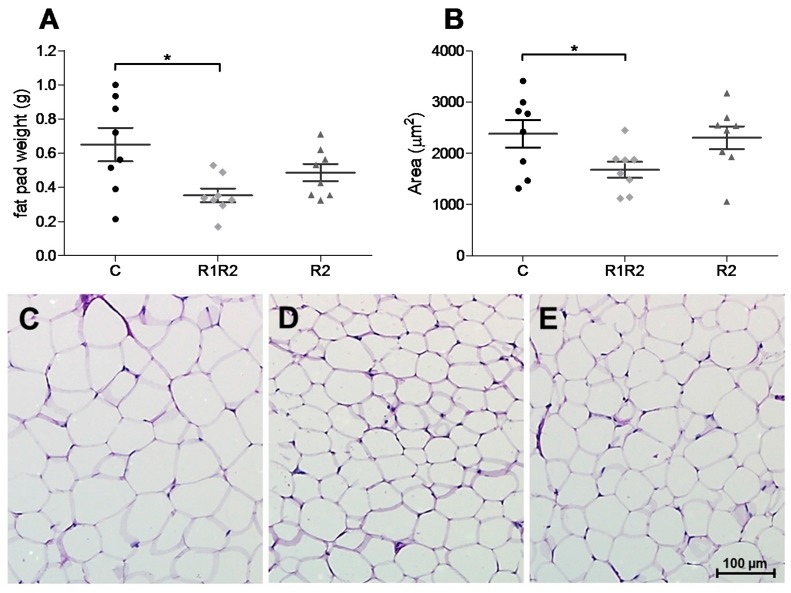
Adipose tissue. Panel (**A**): Wet weight of epididymal white adipose tissue depots after 16 h fasting at dissection (*t* = day 126/128). Significant differences (*p* < 0.05) are indicated with *; Panel (**B**): Epididymal adipocyte size, expressed as average cross sectional surface area of 400 analyzed cells per animal. In panels (**A** and **B**) individual data for each group are shown in different symbols, and mean ± SEM are shown as a horizontal line with error bars. Cell size in R1R2 animals shows a trend for reduction (*p* = 0.0699); Panels (**C**–**E**): Representative images of hematoxylin/PAS stained eWAT of Control (panel (**C**)), R1R2 (panel (**D**)), and R2 (panel (**E**)) animals. The size bar in panel E indicating 100 µm is valid for all three panels.

The differences in BW at the end of the study are reflected in differences in the weights of adipose tissue depots. The wet weight of eWAT is significantly reduced in R1R2 animals (Figure 5). The eWAT in R2 animals seems to be reduced (non-significant lower) compared with controls, but not as much as in R1R2 animals. For pWAT depots a similar effect is observed, with a statistical significance between R1R2 and C animals (R1R2: 0.076 ± 0.014 g; C: 0.197 ± 0.036 g; R2: 0.117 ± 0.021g, *p* < 0.02). The smaller weight of eWAT depots is accompanied by a trend towards a smaller mean cross-sectional area of the adipocytes in the R1R2 animals, compared with controls (*p* = 0.0699). R2 animals have intermediate-sized adipocytes (Figure 5B,C).

#### 3.2.5. Serum Concentrations of Lipids and Peptide Hormones

Serum leptin concentrations at dissection were significantly reduced in BW cycling animals compared with controls, without any differences between the R1R2 and R2. Serum leptin concentrations correlated positively with eWAT mass in control animals, but not in the two BW cycling groups (data not shown). Serum glucose, adiponectin, FFA, and TG concentrations were not significantly different between the experimental groups (Table 2). Serum adiponectin concentrations tended to be increased in R2 animals compared with control animals (1-Way ANOVA *p* = 0.0725). Adiponectin concentrations were not correlated with either BW or eWAT mass (data not shown). Serum TG concentrations tended to be lower in R2 animals compared with controls (1-Way ANOVA *p* = 0.0524, post hoc *p* < 0.05). Serum insulin concentrations were below detection level in all but one sample, and are therefore not taken into account.

## 4. Discussion

Here, we investigated whether a history of BW cycling affects parameters of metabolic health by means of a well-controlled intervention study with an improved setup compared with previous BW-cycling studies, as suggested by Reed and Hill [4]. We excluded effects of changes in dietary composition on the BW cycle, we included an *ad libitum* fed and age-matched control group, and we investigated metabolic health when animals were in a new equilibrium state after BW cycling (i.e., not during recovery). This allowed us to evaluate the effects of repeated BW loss and regain *per se*. We show that one or two BW cycles do not negatively impact established parameters of metabolic health, such as BW gain, food efficiency, energy balance, adiposity, adipocyte size, fasting serum glucose, leptin, adiponectin, TG and FFA concentrations. Furthermore, BW loss during the second period of CR was higher if animals had undergone an earlier BW cycle. These results indicate possible positive effects of CR-induced BW cycling in mice, such as a lower amount of adipose tissue, BW, and leptin concentrations, combined with unaltered serum glucose concentrations.

When comparing our results with other studies, we faced a number of obstacles. Only three publications allowed for a comparison because they induced BW cycles by alternating restriction and *ad libitum* re-feeding on the same diet and allowed animals to regain BW to equilibrium [17,20,21]. In these studies, EE was not measured. Other studies either did not analyze BW regain after BW cycling [11], or did not allow animals to regain BW in their natural way towards a new equilibrium [8,12,22,23], or did not include one-BW cycle control groups [6,8,9,24], or used very short duration cycles and/or total food deprivation [15,16,25], or analyzed only one cycle [14]. In the three studies with which we could compare, BW gain over the total study period was reduced after BW cycling [17,20,21]. Thus, our data confirm the results in previous studies in which BW cycles are induced on the same diet. This suggests that BW cycling *per se*, without changes in diet composition, does not lead to increased BW, nor increased rate in BW gain, nor decreased rate in BW loss.

To determine whether the lower BW after BW cycling was due to changes in the efficiency with which the body handles the consumed energy, we calculated food efficiency (ratio of BW change over food intake) at the end of the study, and found no differences between BW cyclers and control animals (Table 1). Only one of the three studies used for comparison also measured food efficiency; a reduced food efficiency in BW cycling compared with *ad libitum* fed female rats was reported [20]. In the only other BW cycling study performed in mice (of unknown gender), comparable food efficiencies between three cycling groups were observed [22]. Other BW cycling studies, using dietary changes to induce cycles showed no or increased food efficiency [5,7,14] or did not measure this parameter [6,8,10,11,22,26,27]. Based on these data, it seems that BW cycling without dietary changes does not lead to increased food efficiency.

At the end of our study, the energy balance and growth rate were comparable between all three experimental groups. We also observed comparable EE between the three experimental groups, even though the BW cycling animals gained less BW. In addition, eWAT and pWAT masses were reduced in parallel with the BW changes, suggesting that BW cycling mice did not lose lean body mass, but only fat mass, and therefore maintained normal EE. Only three other BW cycling studies measured O_2_ consumption. A lower lean body mass indeed corresponded to lower O_2_ consumption after BW cycling in female rats [26]. Simpson et al. showed higher O_2_ consumption after re-feeding then after BW loss, but did not look for changes during an energy balance state after BW cycling [22]. Schofield et al. analyzed male mice after one weight cycle and found that O_2_ consumption, CO_2_ production, and RER differed depending on the diet in the analysis phase, but did not differ between cycling and non-cycling animals, in agreement with previous data from our group [13]. These studies all induced BW cycles by changing diet compositions, thus, our study is the first to show that BW cycling *per se* on a MFD does not affect long term EE nor energy balance in mice, and leads primarily to a loss of fat mass.

The parallel reductions in BW and adipose tissue wet mass in cycling animals confirm earlier findings [20]. However, adiposity does not always parallel BW changes after BW cycling on the same diet [21]. When BW cycles were induced on a 45 en % high-fat diet, a (not significantly) reduced BW was accompanied by enlarged eWAT mass and adipocyte size, while on a 22 en % MFD, eWAT mass and adipocyte size tended to be reduced in parallel with BW. Another study showed that internal fat increased after a high–low–high fat diet weight cycle, compared with a continuous high fat diet [14]. These results suggest that changes in adiposity as a consequence of weight cycling may depend on the composition of the diet.

Our observations on the concentrations of serum parameters support the conclusion that BW cycling *per se* does not impair metabolic health, usually understood as having (circulating) parameters of metabolism in the normal range. Serum leptin concentrations were significantly reduced after two BW cycles, confirming data in mice and humans [28] and paralleling the reductions in BW and fat mass. The smaller adipocytes in these mice may be secreting less leptin, as has been observed in humans [29]. However, they did not secrete higher concentrations of adiponectin, as could be expected. Adiponectin concentrations are low in obesity and are associated with insulin-resistant states in humans and mice (reviewed in [30]). BW loss often results in higher adiponectin concentrations. It has been shown that fasting acutely reduces adiponectin concentrations in mice [31], and our data are after a 16 h fast. Furthermore, in humans, BW loss must exceed a certain level (i.e., 10% or 12 kg) for adiponectin concentrations to be significantly increased [32]. We cannot exclude that the fasting obscured an effect of BW cycling on serum adiponectin concentrations, although all animals underwent the same fast.

Serum TG and FFA were not significantly affected after BW cycling, confirming previous results showing that TG concentrations will change during BW loss and regain phases of the study, but return to control concentrations thereafter [11,21]. Fasting glucose concentrations were also unaffected by BW cycling, although the glucose concentrations were fairly high for animals that have been fasted overnight. This may be due to the use of anaesthesia [33]. Indeed, glucose concentrations were lower and did not differ between groups (control 7.2 ± 0.22; R1R2 7.8 ± 0.46, R2 7.7 ± 0.47 mmol/L, mean± SEM) when serum samples were taken without anaesthesia by tail-cut before the final fast. Others also reported no changes in glucose concentrations after BW cycling [20,21]. Unfortunately, serum insulin concentrations were too low to be detected faithfully in the present study, thus hampering calculating indicators for insulin resistance, such as the HOMA-IR index. Evaluation of the raw mean fluorescent intensity results of the Milliplex Insulin assay did not show any differences between the three experimental groups (data not presented). Only few studies [20,21,24] measured insulin after weight cycling and observed no significant differences. Altogether, our data show that the metabolic serum parameters FFA, TG, and glucose are not negatively affected by BW cycling. Although our study was above average in length compared with other weight cycling studies, it would be of interest to follow the animals for a longer period of time for further support of our conclusions. In addition, by the inclusion of additional cycles, potential effects on the speed of weight regain could be resolved. Of course, it is important to also validate the findings in females.

## 5. Conclusions

Our study is the first to investigate the effects of BW cycling *per se* on in vivo and ex vivo parameters of metabolic health in one study. All measurements suggest that a history of BW cycling has no adverse effects on metabolic health in male mice. If any effects are found, these rather suggest a lower BW and healthier body composition. Does this result imply that all websites and information on BW cycling and the yo-yo effect in humans should be discarded and removed? An affirmative answer is supported by a recent Japanese study [34] and by a recent review of human studies that concluded that there was no convincing evidence for adverse effects of weight cycling [35]. On the other hand, in humans, associations of adverse changes in body mass index or mortality rate with a higher incidence (or prevalence) of BW cycles or with higher BW loss during BW cycles [2,35,36] have been observed. However, these differences in the outcome could be attributed to other lifestyle changes, in particular, changes in diet composition [34]. Most people, when trying to lose BW, often follow diets with a largely changed macronutrient composition. Our study does not mimic this situation, since we only reduced the amount of calories. Because we are the first to perform such a controlled and complete evaluation of metabolic effects of BW cycling, our findings need to be reproduced and extended to, e.g., establish a possible modifying role of diet composition on the outcome of BW cycling.

## Figures and Tables

**Table 1:** Comparison between *ad libitum* fed control mice, mice undergoing two weight cycles (R1R2) and mice undergoing only the last weight cycle (R2).

Parameter	Control (*n* = 8)	R1R2 (*n* = 8)	R2 (*n* = 8)
BW change *t* = 0–119 (g)	3.63 ± 0.36^ a^	1.95 ± 0.37^ b^	2.68 ± 0.34^ a,b^
FI cumulative (*t* = 0–119) (g)	379.9 ± 4.4^ a^	347.0 ± 4.4 ^b^	361.7± 7.2^ a,b^
BW at *t* = 119 (g)	28.68 ± 0.86 ^a^	26.99 ± 0.52^ a^	27.79 ± 0.67^ a^
FI (*t* = 116–119) (g/day)	2.87 ± 0.13^ a^	2.90 ± 0.07^ a^	2.95 ± 0.12^ a^
FE (*t* = 116–119; BW gain/FI)	0.014 ± 0.023^ a^	0.037 ± 0.026^ a^	0.037 ± 0.023^ a^
EE (kcal/24 h)	11.33 ± 0.08 ^a^	11.09 ± 0.16^ a^	11.17 ± 0.26^ a^
EI (kcal/24 h)	12.63 ± 1.40 ^a^	12.25 ± 0.57^ a^	12.92 ± 0.81^ a^

Body weight (BW), cumulative food intake (FI), BW and FI at end, and food efficiency (FE) are given. Total energy expenditure (EE) and energy intake (EI) data are obtained from the last 24 h of the 48 h of the Calo5 measurement (*t* = day 112–116). These data are the source of the energy balance ratio depicted in Figure 4. Data are mean ± SEM. Different superscript characters (a, b) indicate statistically significant differences (*p* < 0.05).

**Table 2:** Serum concentrations at dissection after 16 h fast (mean ± SEM.).

Parameter	Control	R1R2	R2
Glucose (mmol/L)	12.11 ± 0.72 ^a^	11.88 ± 0.60 ^a^	11.48 ± 0.58 ^a^
Leptin (ng/mL)	2.73 ± 0.64^ a^	1.04 ± 0.36^ b^	1.09 ± 0.15^ b^
Adiponectin (µg/mL)	28.15 ± 2.52^ a^	29.62 ± 2.77^ a^	35.47 ± 1.03^ a,#^
FFA (µmol/L)	793.9 ± 66.1 ^a^	667.5 ± 89.6^ a^	727.6 ± 67.8^ a^
TG (mg/dL)	47.45 ± 2.46^ a^	43.32 ± 2.27^ a^	39.39 ± 1.76^ a,#^

Group size is *n* = 8 for all parameters and groups, except for serum leptin levels in control animals (*n* = 7). FFA are serum free fatty acids and TG are serum triglycerides. Different superscript characters (a, b) indicate statistically significant differences (*p* < 0.05). ^#^ indicates a trend (Adiponectin *p* = 0.0725; TG *p* = 0.0524 (posthoc C vs. R2: *p* < 0.05)) towards different concentrations compared with control animals.
